# Self-assembled peptide amphiphiles function as multivalent binder with increased hemagglutinin affinity

**DOI:** 10.1186/1472-6750-13-51

**Published:** 2013-06-18

**Authors:** Christine Hüttl, Cornelia Hettrich, Reinhard Miller, Bernd-Reiner Paulke, Petra Henklein, Harshadrai Rawel, Frank F Bier

**Affiliations:** 1 Fraunhofer Institute for Biomedical Engineering IBMT, Am Mühlenberg 13, 14476, Potsdam, Germany; 2 Institute for Biochemistry und Biology, University of Potsdam, Maulbeerallee 2, 14469, Potsdam, Germany; 3 Max Planck Institute for Colloids and Interfaces, Am Mühlenberg 1, 14476, Potsdam, Germany; 4 Fraunhofer Institute for Applied Polymer Research IAP, Geiselbergstr. 69, 14476, Potsdam, Germany; 5 Institute for Biochemistry, Charité – Universitätsmedizin Berlin, Virchowweg 6, 10117, Berlin, Germany; 6 Institute of Nutritional Sciences, University of Potsdam, Arthur-Scheunert-Allee 114-116, 14558, Nuthetal, Germany

**Keywords:** CMC, Influenza virus detection, Micelle, PAs, Surface plasmon resonance

## Abstract

**Background:**

A promising way in diagnostic and therapeutic applications is the development of peptide amphiphiles (PAs). Peptides with a palmitic acid alkylchain were designed and characterized to study the effect of the structure modifications on self-assembling capabilities and the multiple binding capacity to hemagglutinin (HA), the surface protein of influenza virus type A. The peptide amphiphiles consists of a hydrophilic headgroup with a biological functionality of the peptide sequence and a chemically conjugated hydrophobic tail. In solution they self-assemble easily to micelles with a hydrophobic core surrounded by a closely packed peptide-shell.

**Results:**

In this study the effect of a multiple peptide binding partner to the receptor binding site of HA could be determined with surface plasmon resonance measurements. The applied modification of the peptides causes signal amplification in relationship to the unmodified peptide wherein the high constant specificity persists. The molecular assembly of the peptides was characterized by the determination of critical micelle concentration (CMC) with concentration of 10^-5^ M and the colloidal size distribution.

**Conclusion:**

The modification of the physico-chemical parameters by producing peptide amphiphiles form monomeric structures which enhances the binding affinity and allows a better examination of the interaction with the virus surface protein hemagglutinin.

## Background

Specific binders to the virus surface protein hemagglutinin (HA) are of interest for influenza diagnosis as well as for therapy. Besides sialylglycoconjugates and antibodies [1-3], sialic acid mimicking peptides have also the potential as high affinity binders [4]. In the present study, we used the advantages of peptide amphiphiles (PAs) for influenza virus detection, in particular for the H5N1 virus. Influenza A virus subtype H5N1, commonly called “bird flu”, is a highly pathogenic avian influenza virus with high death rates - 100% mortality of the birds within 48 hours [5]. It was first detected in 1997 in Guangdong (China) [6,7]. The virus does not only infect a large number of poultry but also increasing numbers of human beings, often with fatal consequences.

The influenza virus infection occurs by an attachment of its surface protein hemagglutinin to the host cell. The protein represents a homotrimeric lectin and appears at high concentrations on the surface of the virus, with 600–1200 molecules per virus particle [8]. Furthermore, the HA is also responsible for the viral binding, allowing the infiltration into the host cell through endocytosis and subsequently the fusion of the membranes. HA binds to the sialic acid (SA) terminal residues of glycoproteins and lipids of the host cell surface. The interaction consists of a multivalent binding [9] to gain stability and to prevent the dissociation of the virus. The association constant for the single SA molecule to a single HA receptor is relatively weak with 10^3^ M^-1^, but the binding between viral receptor and cellular SA increases substantially as characterized by a multivalent affinity constant of estimated 10^13^ M^-1^[8,10]. The receptor binding site describes a pocket like cavity and is located on the distal end of the molecule [11], whereby this region is known to be highly conserved in all strains of the virus [12].

Matsubara et al. [4] researched for an alternative of sialic acid in order to inhibit the internalization of H1N1 and H3N2 influenza virus into host cells and their efforts were rewarded with a minimum peptide sequence ARLPR showing a good inhibitory activity (IC_50_ = 1.9 μM for H1N1 and 1.6 μM for H3N2). A docking simulation involving the proposed minimum sequence in the receptor binding pocket of HA showed, that the side chains of the amino acids of the peptide utilized the same hydrogen bonds and van-der-Waals interactions to HA as the natural binder sialic acid [4].

Via solid phase peptide synthesis (SPPS) [13,14] it is possible to efficiently produce peptides with corresponding high purity. This approach provides a precise control over the molecular structure and enables simultaneously an easy manipulation by chemical modification. In the concept of this study, functional peptides were designed as more potent peptide ligands with an N-terminal alkyloyl chain, which in turn promotes a self-assembly into highly ordered structures in aqueous phase. Thus modified peptides exhibit the morphology and behaviour of peptide amphiphiles and hence the combined characteristics of both - the biological activity of the peptides as well as the properties of surfactants such as CTAB (hexadecyl-trimetylammonium bromide) or SDS (sodium dodecyl sulphate). Peptide amphiphiles are built up of a hydrophobic segment, generally a single- [15,16] or a double-alkyl tail [17], and a hydrophilic biologically active peptide headgroup with the capability of self-assembly. The hydrophobic chains allow the monomers to form molecular aggregates in solution based on the hydrophobic interactions between the hydrocarbon chains. These aggregate states can emulate the folding of the peptides simulating their native secondary structure. It can be visualized as an interaction between the hydrocarbon chains in the core and the resulting complementary alignment of the peptides in the corona as described by Israelachvili et al. [18]. The PA building blocks arrange in well-defined and reproducible structures such as micelles, bilayers, vesicles, spheres or cylinders with the peptides oriented in an organized and functional way. Their applications range from diagnostic tools [19] over therapeutic approaches [20,21], drug delivery systems [22] to functional biomaterials [23]. With this molecular set-up the penetration through cell membranes is much better and the accessibility of the peptides to receptors enhanced.

To analyse the specific interactions between HA and the peptide amphiphiles we developed a rapid and highly sensitive method using a surface plasmon resonance system (SPR). This label-free and real-time optical detection method is a well established characterisation module [24,25] for biomolecular interactions of proteins [26], oligosaccharides, pathogens [27,28], cells and lipids up to smaller molecules. SPR-based measurements are sensitive to refractive index changes and correlates with the mass of the immobilised ligand. The surface coverage by binding is measured in resonance units (RU), the signal follows the binding in real-time during the association and dissociation process. The SPR measurements provides detailed insights into the mechanism of the interaction between the peptide amphiphile micelles as multivalent ligands and the HA receptor. The sensor surface could be regenerated and allows multiple use of the same chip for subsequent analyses with a good repeatability.

These advantages of the SPR technique enable us to analyse the kinetics of the interaction of the soluble PAs to the hemagglutinin of the pathogen H5N1 (Table 1). In addition, to evaluate the properties of the newly developed PAs, for instance the critical micelle concentration (CMC) or colloidal size, could be determined. Surfactants start to form micelles from a critical concentration on, called CMC. For the identification of the CMC, different physicochemical characteristics can be used considering the PA concentration. There are several properties of surfactant solutions which exhibit a break point in the concentration dependence: electric conductivity, osmotic pressure, interfacial tension, turbidity or surface tension [29]. In this study surface tension measurements were utilized to determine the CMC.

**Table 1:** The amino acid sequence and the molecular weight of the used peptides

**Peptides**	**Sequence**	**Mw [g/mol]**
*modified peptides*		
Pal L1	C_16_-ARLPRTMVHPKPAQP	1937
Pal M1	C_16_-ARLPRTMV	1181
Pal S1	C_16_-ARLPR	849
negative control	C_16_-GSWGEW	957
*unmodified peptides*		
L1	ARLPRTMVHPKPAQP	1698
M1	ARLPRTMV	942
S1	ARLPR	611
negative control	GSWGEW	720

Besides the concentration of micelles, size plays also an important role. Light scattering methods such as static light scattering (SLS) and dynamic light scattering (DLS) are the most efficient techniques of micelle size and shape measurement. The DLS has verified to be a rapid and precise method for sizing particles in a nanometre range [30]. The technique is based on time-resolved analysis of the Brownian motion, small molecules moving faster than large molecules, which causes a Doppler shift of the incident laser light frequency.

In the present study biophysical methods such as SPR will be used to examine the effect of micelle formation of palmitoyl peptides with binding to the receptor binding site of the virus surface protein HA.

## Results and discussion

### Peptide amphiphile synthesis

**Figure 1:**
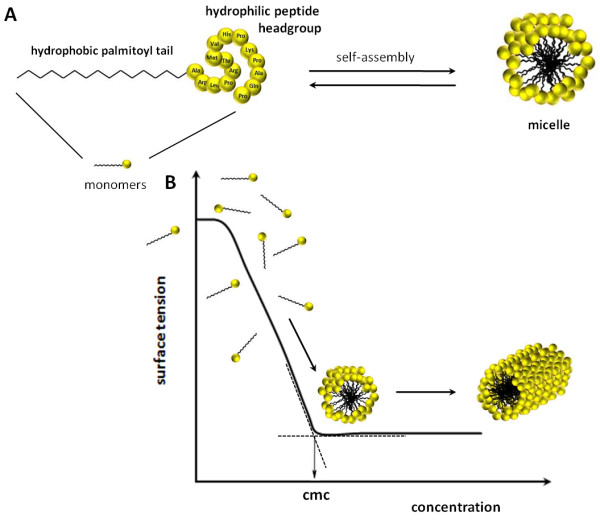
**Characteristic curve of the surface tension for aqueous surfactants solution. A**) schematic presentation of the peptide amphiphile with the hydrophobic C_16_-alkylchain and the hydrophilic peptide headgroup forming micelles by self-assembly above the critical micelle concentration (CMC). **B**) idealised curve of surface tension as a function of surfactant concentration with the CMC representing the point, where surfactant molecules start to form aggregates known as spherical micelles, with further increasing concentration, the micelles change the shape to cylindric forms.

Our initial investigations showed that the binding between hemagglutinin (HA) and the unmodified peptides (S1, M1 and L1), which were selected from literature [4], is very difficult to determine. The response signals were barely distinguishable from the background and the evaluation was almost impossible. Thus, a method is required to enhance the signal and its sensitivity. The solution seems to be the use of an amphiphilic peptide with a polyvalent display of biofunctional peptides [31]. Matsubara et al. [4] did some cell tests with *N*-stearoyl peptides (C_18_-peptides) with excellent inhibition effects up to 90 percent. In Figure 1A a schematic presentation of our PA's and their micellar character is illustrated. To identify which sequence length of the peptide is sufficient for our SPR studies the 15mer L1 and the 8mer M1 as a middle sized ligand were utilised in addition to the required 5mer minimum sequence S1 (Table 1). Different N-terminal modifications were performed to identify the best possible ligand for HA. A PEGylation of the peptides or a dimeric structure showed no improvements in the binding activity (data not illustrated). The modification with a palmitic acid residue has been reported to indicate promising results in this context [4,8,31]. Using the solid phase peptide synthesis (SPPS), the peptide sequence was built up stepwise and the preactivated palmitic acid was coupled in the last step to the N-terminus of the peptide sequence. The conjugated fatty acid supplies the hydrophobic driving force for a self-assembly in solution. The three synthesized palmitoyl peptides are shown in Table 1.

To analyse the effect of the modification for the HA binding behaviour, the hemagglutinin H5 was immobilised as ligand to the SPR sensor chip surface.

### SPR measurements

### Immobilisation of HA from H5N1

The HA (H5N1) was immobilised on an activated carboxymethylated (CM)-dextran sensor surface via the Biacore® standard activation protocol [32] and in connection with a pH 4 immobilisation buffer. A low HA concentration in combination with a longer injection time was more efficient than higher concentrations at lower injection times. Otherwise the individual HA’s would interfere each other and no adequate loading of the surface can be observed. Under these conditions, about 6400 response units (RU) could be achieved from the immobilised ligand to the flow cell. As control for the HA immobilisation, the monoclonal H5N1 antibody was used. At a concentration of 1.0 μg/ml, a distinct response signal at the binding flow cell was detected. The immobilisation of HA was successful and the accessibility of hemagglutinin was warranted.

### Palmitoyl peptides as multivalent binding partner

**Figure 2:**
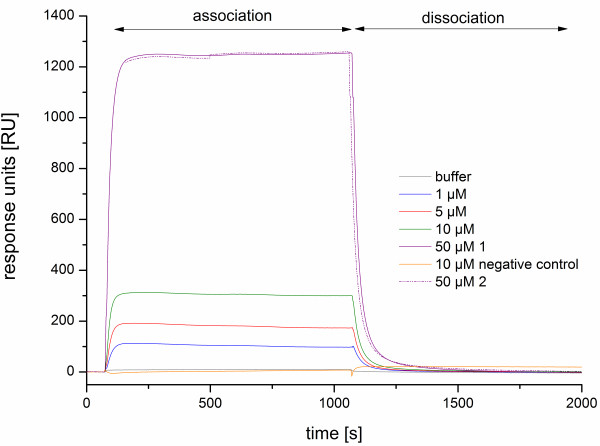
**SPR measurements of Pal M1 to H5 modified sensor chip surface.** An overlay plot illustrating the determination of the modified peptide Pal M1 at increasing concentration displaying association and dissociation processes and the observed leap between 10 and 50 μM in HBSP buffer solution. As a control, the negative probe with no binding affinity to H5 is shown. The reproducibility of the method is illustrated with the 50 μM concentration (1+2).

**Figure 3:**
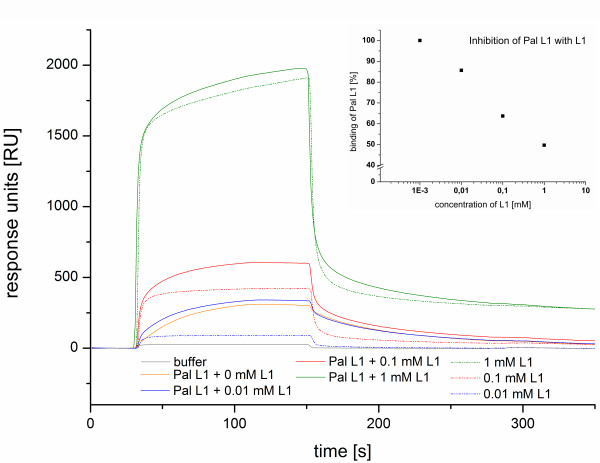
**An overlay of sensorgrams by the inhibition experiment of peptide amphiphile Pal L1.** With increasing concentration of the corresponding unmodified peptide L1 and immobilised H5 on the biosensor surface could be observed an inhibition of Pal L1 (with a constant concentration of 5 μM). Up to 50% inhibition at a concentration of 1 mM L1 (top curves, response signal 170 s after the cycle starts) occurs. The insert shows the linear behaviour of the inhibition depending on the concentration of the peptide L1.

To obtain more data on the peptide - hemagglutinin interaction, the Biacore instrumentation provided the most suitable “real-time biospecific interaction” analysis platform [33]. The binding experiments were performed with an increasing PA concentration and with the various peptide sequences. The unmodified peptides and the PA's were successively injected and analysed by Biacore evaluation software and reproducible binding results for three times were observed (Figure 2, 50 μM 1 and 2). Both, the peptides and PAs offered a expected binding profile of a SPR measurement with the association process, where the peptides and micelles interact with the immobilised hemagglutinin. This results in a characteristic binding equilibrium, which generally approaches a plateau. The peptides have the characteristic feature of being rather weak binders with a low-affinity interaction (K_D_ of 1 mM from previous experiments with the unmodified peptides) and may dissociate quickly. The sensorgram of the dissociation process for the unmodified peptide L1 is shown in Figure 3. A regeneration step was not necessary. In contrast, the control flow cell, where no HA was immobilised, showed no significant signal changes (data not illustrated).

**Figure 4:**
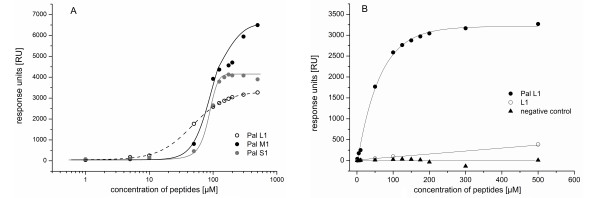
**The plotted interaction curves of the PAs against H5. A**) The compared binding curves of Pal L1, M1 and S1 in response to the PA concentration. **B**) The binding curve of Pal L1 and the unmodified L1 with the negative control (also palmitoyl end with a non-binding peptide sequence) as a function of peptide concentration. All data have been corrected with corresponding blanks.

Figure 2 shows the dependence of the sensor signal (response units) on concentration. The high increase in signal response from 10 to 50 μM peptide concentration is remarkable, this couldn't be observed by the unmodified peptides. Also in the next figure (Figure 4A) this concentration dependence is given for the three different peptide amphiphiles. Slight signals were detected in the lower concentration range of 1 to 10 μM. When the concentration of PA's was augmented to 50 μM, a sharp increase in the response units was recorded. The signals moved forward to saturation at 200 μM (Figure 4A, Pal L1 and Pal S1), whereas Pal M1 still slightly rose at this concentration and has not yet reached its maximum. The binding of Pal M1 in the upper concentration range was difficult to evaluate and measurements above the concentration of 500 μM peptide were necessary.

Pal L1 initially achieved constant response units at about 3000 RU. This peptide has the biggest head group and shows a characteristic binding curve. The curves of Pal M1 and Pal S1 are distinguished by a steep slope. The starting concentration for the binding is in correlation to the micelle formation (see below). Pal L1 has a small CMC and began binding to HA at the lowest concentration. The binding of HA requires higher concentrations of Pal M1 and Pal S1 to initiate this process.

Figure 4B shows the binding curve of the unmodified peptide L1 in comparison to the palmitoyl peptide Pal L1. At a concentration of 500 μM, the signals of Pal L1 were about tenfold higher than the unmodified one. As a control, we also designed a PA with no binding activity to HA, but with the capability to assemble into micelles. This negative control showed no quantitative measurable signal changes (Figure 4B and compare also the sensorgram in Figure 2) and thereby demonstrated the specificity of the tested PA's. In addition, the binding capacities of M1 and S1 were comparably lower (also not illustrated in Figure 4B for the sake of clarity). These small affinities of the unmodified peptides were to be expected. Chang and co-worker observed that linear peptides are low-affinity ligands, which do not have a distinct conformation in solution. The corresponding cyclic peptides bound 1000-fold firmer than their linear counterpart [34], because the peptides were constrained by cyclisation and lose their conformational freedom. Correspondingly, the entropy of such unbound peptides is reduced [34,35]. This entropy decrease is also achieved by an arrangement into a micelle. It enhances the binding affinity to HA and shows high binding signals in the sensorgrams. This PA arrangement can be used as diagnostic tool similar to Guler et al. [36], where they designed a biotinylated peptide amphiphile for enhanced recognition by the avidin receptor or where Bull et al. [37] utilized, a peptide amphiphile to raise the relaxivity of magnetic resonance imaging agents. Different peptide amphiphile constellations represent a promising way of functionalising biomaterial interfaces.

The calculation of the kinetic data is rather difficult, due to the multivalent binding mode of assembled micelles [19]. It is not known, how many free binding sites of peptides are available at the surface of each micelle. So far, there is no satisfactory description of the binding, only some promising approaches could be found in literature [38,39]. Therefore, further investigations and evaluations seem to be necessary.

### Inhibition test

To demonstrate the specificity of the micelle binding, a competitive inhibition assay was performed by using the unmodified peptide in excess. The PA was added at constant concentration of 5 μM to a mixture with rising quantity of the corresponding unmodified peptide. This resulted in a competition for the same binding site. The corresponding SPR sensorgrams are shown in Figure 3. The binding of Pal L1 to the H5-modified sensor surface is described by a typical binding curve (orange curve). In the presence of 0.01 mM peptide L1 an enhancement was detectable for the binding of L1. With an excess of L1 the maximum response rose again, but without significant differences to the signal without Pal L1. The results clearly show that an inhibition of Pal L1 is possible. At a concentration of 1 mM L1 the binding capacity is up to 50 percent lower (see inset of Figure 3). The interaction to H5 can be analysed by applying PA and the specificity of the binding was not affected by self-assembly.

For a more precise characterisation of the properties of the new PAs, the micelle size, shape and micelle building concentration were determined.

### Micelle characterization

The incorporation of a long alkyl chain to the peptides resulted in a surfactant like behaviour. It allows even peptides that normally do not self-assemble to form stable aggregates. Not only self-assembly can be induced via the attachment of alkyl-tails, also the orientation of the secondary structure can be changed for stabilising a peptide in its bioactive conformation [40].

The aggregation behaviour of amphiphilic peptides in aqueous solution was determined by surface tension measurements. A characteristic curve of the surface tension as a function of the surfactant concentration is illustrated in Figure 1B. In aqueous solution, the palmitoyl peptide molecules are present as monomers and form a monolayer film at the air-water interface. With increasing PA concentration, the monolayer adsorption becomes complete and an abrupt change indicates the critical micelle concentration (CMC) of the PA. At this point, the formation of thermodynamically stable aggregates, called micelles, starts. Concentrations above the CMC result in constant surface tension values according to the adsorption-isotherm of Gibbs. Any increase in the peptide concentration does not affect the number of monomers, but influences the number and shape of the micelles [41], and the transformation into bigger lamellar, hexagonal or cubic assemblies.

**Figure 5:**
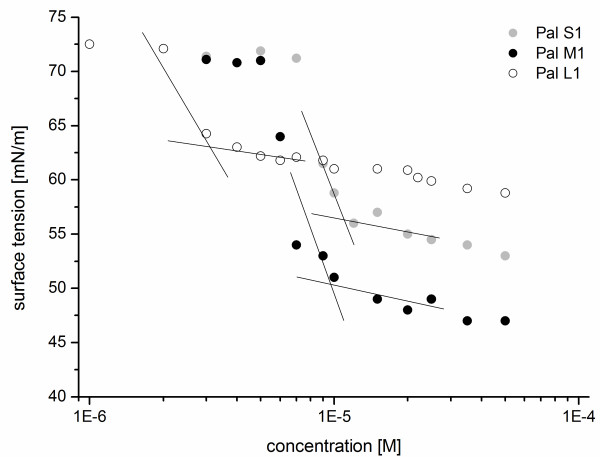
**Pendant drop measurements.** The surface tension isotherms of peptide amphiphiles Pal S1, Pal M1 and Pal L1 in HBS buffer as a function of peptide concentration.

In the present work the equilibrium surface tension of the PA solution was analysed by a drop profile analysis tensiometer at different concentrations. Figure 5 shows the equilibrium surface tension in dependence on PA concentration. The surface tension decreases from 72.8 mN/m (surface tension of pure water) with increasing PA concentration and remains constant from a certain concentration on at a surface tension of 50 mN/m. This distinctive concentration is the above-mentioned critical micelle concentration of the PA and initiates the aggregation into micelles.

**Figure 6:**
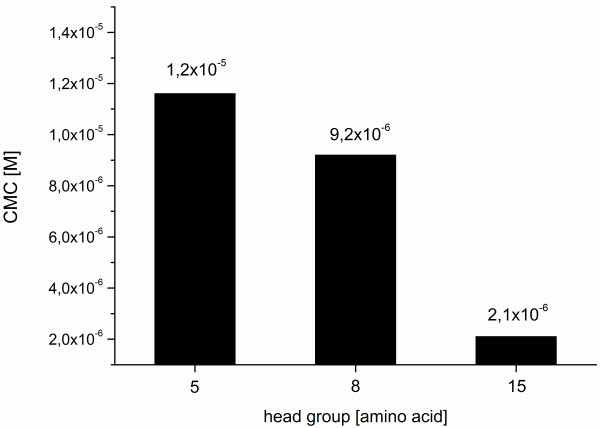
**CMCs of the peptide amphiphiles.** The values of critical micelle concentration depending on the headgroup size of the peptide amphiphiles Pal S1, M1 and L1.

The CMC value is determined by a linear fitting of the two data subsets and calculating the concentration at their intersection. The surface tension data correlate to the behaviour of an ionic surfactant with a CMC of 10^-6^ to 10^-5^ M. The values of Pal S1, Pal M1 and Pal L1 do not really delineate to a straight line beyond the CMC - the surface tension further on slightly decreases. This means an indication of the commencing of the aggregation to bigger micelles, rods or disks like the cylindric form in Figure 1B. Further investigations by cyro-TEM micrographs could give more informations about the morphology of the micelles. The CMC values (compare Figure 6) are conform with the SPR measurements. This becomes apparent for example in the strong increase in binding signal between 10 and 50 μM (Figure 2), which precisely matches the CMC value with 10 μM. At this stage, the micelle formation begins and a multivalent binder is available for the HA interaction. The signal increases and the micelles grow further. At a certain point, no more micelles are formed and a shape change can be observed, which is indicated by the slight decrease of surface tension at higher concentrations (Figure 5) and the saturation level as depicted in the SPR binding curves (Figure 4A).

Similar to Buckingham et al. [42], the CMC of the PA's depends also on the size of the head group and decreases with increasing size of the peptide sequence (Figure 6). Probably the behaviour is also controlled by the hydrophobic part, as the CMC decreases with longer chains. This is caused by the tendency of hiding the hydrophobic parts of surfactant molecule in an aqueous environment [42]. With increasing peptide length the hydrophobicity increases and more peptide bonds are available for hydrogen bonding.

**Figure 7:**
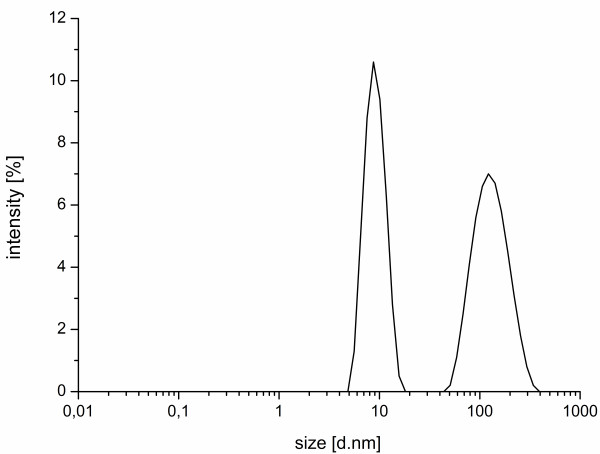
**Colloidal size of the micelle.** The DLS data showing the average distribution of the hydrodynamic radii of micelle Pal L1 at a concentration of 100 μM in HBS buffer solution with a polydispersity index of 0.3.

The HA trimers are located on the surface of the influenza virus with a distance of 12 nm. The individual receptors of the monomers are separated 4 nm from each other [43]. Dynamic light scattering (DLS) measurements were carried out to provide information about the shape and size of the PA micelles, to clarify whether the PAs are large enough for the described binding distances. The determination was done at a concentration of 100 μM above the respective CMC. The size distribution showed colloidal structures of 7 nm in diameter and a broader peak between 50 and 200 nm (Figure 7). With this result, the PAs supply excellent counterparts for the HA detection. A diameter of 7 nm is sufficient for the receptor binding site. Ideally one micelle could bind to one HA trimer, where the receptor binding sites of each monomer have a distance of 4 nm. The larger micelles have the ability to connect with several HA trimers.

## Conclusion

In summary, the present work successfully describes the interaction between short peptides and virus surface protein with a SPR binding method. The biological activity and specificity of the peptides could be increased by N-terminal modification. In solution, these modified peptides showed amphiphile behaviour and formed micelles. With such structural constellation, multiple binding sites for improved affinities are available, resulting in corresponding amplified detection signals. It is therefore possible to utilise peptides efficiently in a controlled environment, while considering the values of the critical micelle concentration and the size distribution of the peptide amphiphile association structures. Peptides - arranged in such a controlled micellar alignment - provide the possibility of interaction with multiple binding partners. Prospective applications could be the use as diagnostic tool for protein recognition in combination with a dye-based detection system. Upon successful binding of the micelle and the consequent release of the dye from inside of the micelle would be provided an elegant strategy to monitor the interaction.

## Methods

### Chemicals

#### Peptide synthesis

The peptides and palmitoyl peptides (Pal L1, M1 and S1) were prepared on a rink amide-chemmatrix resin (PCAS BioMatrix Inc, Canada, loading 0.47 mmol/g). The peptides were synthesized by an automated peptide synthesizer using 9-fluorenylmethyloxycarbonyl (Fmoc) chemistry. The palmitic acid was coupled over the activated carbonyl group at the N-terminus of the peptides Pal L1, M1 and S1. Following cleavage from the resin with 95% trifluoroacetic acid (TFA), 2,5% triisopropylsilane (Merck Schuchardt OHG, Germany) and 2,5% water, the peptides were purified by reverse phase HPLC and verified by mass spectrometry. The purity was greater than 95%.

#### Hemagglutinin sample

The hemagglutinin from influenza A virus is a recombinant protein H5 (HA1 aa 17–338, A/chicken/Jilin/9/2004 (H5N1), AAT76166) and was purchased from ProSci Incorporated (Poway, USA). The corresponding monoclonal antibody H5N1 HA1 (clone 4E10C10) was also aquired from ProSci Incorporated.

#### Buffers and solutions

As the running and dilution buffer, a HBSP buffer consisting of a 10 mM HEPES solution with 150 mM sodium chloride and 0.05% Tween 20 at pH 7.4 was applied. Acetate buffer (10 mM) at pH 4.0 was most suitable for the immobilisation. All buffers were prepared with deionized water and filtered by a 0.2 μm pore mixed cellulose ester (ME 24) Whatman® filter. The surface activation was done with a mixture of 0.4 M *N*-ethyl-*N'*-(3-dimethylaminopropyl) carbondiimde (EDC) and 0.1 M *N*-hydroxysuccinimide (NHS) (Sigma-Aldrich GmbH, Germany) in deion. water. Afterwards the saturation reagent was 1 M ethanolamine-hydrochloride (AppliChem GmbH, Germany) solution at pH 8.5.

### SPR instrument

The Biacore T100 system was used for all binding experiments (Biacore, GE Healthcare, Sweden) with the standard carboxymethylated dextran-coated gold sensor chip CM5 (GE Healthcare, Europe). The operating parameters were a flow rate of 10 μl/min and a running temperature of 25°C. The binding of the peptides to the HA modified sensor surface was expressed in response units (RU).

### Biofunctionalization of the SPR chip with the virus surface protein hemagglutinin

For the assay, the HA protein was covalently immobilised via the standard amine-coupling procedure. After conditioning of the sensor chip with HBSP, the carboxylic groups of the sensor chip were activated by a 1:1 mixture (v/v) of aqueous solution of NHS and EDC for 420 s. After a 120 s break a solution of HA (37.5 μg/ml at pH 4.0) was flowed for 600 s to the channel enabling high ligand intensity. In the following step, the remaining active groups were capped by passing the aqueous ethanolamine solution for 420 s. The control channel was treated in the same way but without HA immobilisation. The specific monoclonal H5N1 antibody was used to examine the extent of H5 immobilisation. The antibody was injected at different concentrations of 0.01, 0.1, 1.0 and 10.0 μg/ml.

### SPR protocol for multivalent binding events

As analyte the PAs with an N-terminal palmitic acid tail were diluted with the running buffer HBSP to a final concentration of 1.0 to 500.0 μM. Within 1000 s, the association of the peptide in solution was done at a flow rate of 5 μl/min and a subsequent dissociation step of 1000 s was performed. Every binding step was also performed over the control channel and using control injections of buffer. NP1 with and without the palmitoyl tail were employed as control peptides without specific binding. The SPR signals were corrected by subtracting the signal measured in the control cell (1, no HA immobilisation) from the signal of the binding cells (2, 3, 4).

### Verification of specific binding to the receptor binding site

The corresponding unmodified peptide (which is known as binding partner for HA Matsubara 2010) was used as inhibitor. A mixture of constant 5.0 μM PA with the unmodified peptide concentrations from 0.01 to 1 mM in excess were injected at a flow rate of 10 μl/min. The SPR signal of each concentration was registered after a contact time of 120 s. The dissociation phase of 120 s was followed by a stabilization period of 300 s. Every binding step was performed over all flow cells at a flow rate of 10 μl/min.

### Evaluation of the SPR data

Multiple sensorgrams for different concentrations of the peptides were corrected, overlaid and aligned by the Biacore T100 evaluation software (version 1.0). The results were subtracted by the control channel to eliminate non-specific binding.

### Pendant-drop method

The surface tension of HBS-buffered peptide solutions was measured until equilibrium was adjusted, using a profile analysis tensiometer PAT-1 (Sinterface Technologies, Germany) with an accuracy of ±0.1 mN/m at T = 22°C. The solution drops were formed with a volume of 15 μl at the tip of a steel capillary. The droplet images were recorded via a CCD camera and transferred by a framegrabber into a PC. There, the drop profile coordinates were extracted and the profile analysed by the Sinterface intern software (calculation of the surface tension by a best fit algorithm of the Gauss Laplace equation to the experimental shape coordinates). The calibration was done with a gauged steel ball.

### Dynamic light scattering

The dynamic light scattering data were accumulated at a scattering angle of θ = 173° (backscattering detection) with a high-performance particle sizer (HPPS-ET, Malvern Instruments, UK), equipped with a helium neon laser (λ = 633 nm) and a thermoelectric Peltier element for temperature control. Autocorrelation functions were analysed with the CONTIN method. Apparent hydrodynamic diameters were calculated according to the Stokes-Einstein equation. All measurements were obtained at 25°C with a 10 × 10 mm polystyrene cuvette. Five measurements, each for 30 seconds, were performed for the individual samples and the values were averaged.

Reference measurements were carried out at 'Particle Size Analysator BI 90' (Brookhaven Instruments, USA) with a 35 mW helium neon laser and a detection angle of 90°.

## Competing interest

The authors declare that they have no competing interests.

## Authors’ contributions

ChH developed the peptide amphihpiles for hemagglutinin detection, carried out the SPR measurements and participated the micelle characterisation and wrote the manuscript; CH was responsible for the conceptual design and coordination of this project; RM performed pendant drop measurements and CMC determination; BRP carried out dynamic light scattering; PH synthesized and purified the peptides; HR revised the paper; FFB supervised the project. All authors read and approved the final manuscript.
